# The Surgical Treatment of Tuberculous Glands

**Published:** 1909-04-03

**Authors:** Arthur Edmunds

**Affiliations:** Surgeon to the Great Northern Central Hospital, Surgeon to Out-Patients, Paddington Green Children's Hospital


### Hospital Clinics.

### THE SURGICAL TREATMENT OF TUBERCULOUS GLANDS.

By ARTHUR EDMUNDS, M.S., F.R.C.S., Surgeon to the Great Northern Central Hospital, Surgeon to Out-Patients, Paddington Green Children's Hospital.

(Abstract of a lecture delivered at the Polyclinic )

The title of my lecture is perhaps rather anomalous, for it presupposes that the surgeon has the diagnosis ready made for him, which is very far from being the case in practice. One would often unhesitatingly advise excision of chronically inflamed or enlarged glands if one knew for certain that they were tuberculous; but this is just the difficulty.

A very usual sort of case to meet with is such as this. The patient is a child or adolescent who is brought to the surgeon because of the slow growth of some glands, most often behind and below the angle of the lower jaw. Interference with the general health has been so slight as to remain long unnoticed, or there may be a story that the patient has always been "delicate." Such a case is by no means diagnostic of a tuberculous affection of the glands, and, although various methods of diagnosis such as Calmette's and the tuberculin reactions have been introduced, it is safe to say they have not yet obtained general recognition; nor in these cases is it often necessary to employ them.

### Operative or Non-operative Treatment.

The problem we have to consider then is how to treat a patient with slight ill-health and some lumps in the neck. I make it a rule in every such case to examine carefully the whole body: first, the other side of the neck, then the axillæ, groins, and the chest and abdomen. As a rule the man who examines the neck only, or but one side of it, often makes no serious mistake and goes unpunished; but occasionally one comes across an enlarged spleen, abdominal glands, or other conditions having an important bearing on the diagnosis. And I will mention one point more now. When I have completed an operation for cervical enlarged glands, I always examine the abdomen for enlarged glands carefully while the patient is still under the influence of the anæsthetic.

One more word of warning. One frequently sees allusion to the treatment of the single enlarged gland. I am almost inclined to doubt the existence of such a pathological condition, yet it is frequently described, and many people believe that it can be shelled out through a half-inch incision.

In considering which cases to submit to operation one has to look upon the enlargement not as a local foreign body, but as a manifestation of a pathological process. In the case of an acute infection of the tissues there is a fight to a finish between the glands and the micro-organisms. Here the contest is short and sharp. But in the case of tuberculosis we are dealing with a much more drawn-out struggle. The issue of the conflict is often doubtful for years, and in the end a large glandular area may be involved. It is possible to fortify the resistance of the body by hygienic measures, and surgical interference must always be considered both in its effects on the parasite and the host. In acute infections such interference may be bad practice inasmuch as it interferes with the proper function of the lymphatic glands. In tuberculosis it may be good practice, for the glands have then fulfilled their functions as protective organs by isolating the invading parasite and confining them within the glands which can be safely sacrificed for the benefit of the rest of the body.

In coming to more practical details, I think it would be well to consider how to deal with a case such as the one we presupposed—a child or young adult with a few small glands which have caused no pain and have only slightly affected the general health.

On examination there are one or more lumps to be felt on one side of the neck; the other side of the neck is clear, the axillary glands are barely, if at all, palpable, and no others are to be found anywhere. In such a case one would be justified in advising an outdoor life in fresh air, and any other hygienic measures that seem suitable, together with the administration of the iodide of iron, which is, in my opinion, definitely the best drug for these patients. But before advising this we must think of the portal of infection. The tonsils, naso-pharynx, floor of the mouth, and teeth must be examined. There are undoubted cases in which adenoid vegetations are the seats of definite tuberculosis, and many more in which the tubercle bacillus has been found in them. Without attaching too much importance to the latter, for the ubiquitous bacillus can be found anywhere that dust can penetrate if a sufficiently careful search is made, there is no doubt that the tubercle bacillus can pass through a mucous membrane without causing any local manifestation of disease, and be deposited in some corner of a lymphatic gland draining that area. It is of little use, therefore, to send a child away without attending first to enlarged tonsils, adenoids, dental caries, etc.

Let us assume next that the glands do not subside, and that they are reasonably well supposed to be tuberculous. The question then becomes one not of whether, but of when, to operate. A great objection on the part of the patient and the friends to operation is the cosmetic one; but it is to be remembered that the practice of waiting for an abscess to form is absolutely certain to result in unsightly scarring, whereas by operating early one may get a neat unnoticeable linear scar.

### Technique of the Operation.

Having decided, then, to operate before the glands have broken down, the first problem that presents itself is that of the incision. We must have one which will enable us to get access to the whole of the disease, which is always more extensive than the lump or lumps that can be felt There are three main types of incision. One is a straight cut along the anterior border of the sternomastoid. This, I think, the worst possible; though it is very commonly employed, it does not follow the natural plane of cleavage of the skin, which runs more or less transversely. The second is an incision by which a large square flap is turned forwards: in some cases this is a great advantage, but as a rule in tuberculous cases it is unnecessary. The scar of the flap incision, extensive though it is, is very little noticed afterwards, but the centre part of it is still at right angles to the natural folds. The third and best type is an almost transverse incision, by which also there is much less liability to injure the fibres of the branches of the facial nerve. Moreover, the fibres of the platysma myoides are cut more directly across, which facilitates the subsequent suturing of this muscle.

After making this incision, one can retract the skin a little, and then divide the platysma. Next, deepen the incision until the sternomastoid is recognised. Seek for the anterior border of this muscle, and then deepen the wound down to the deep fascia. Make an incision deliberately into this, remembering that the jugular vein is beneath it. Having thus turned the flank of the glands by getting behind them it will now be possible to clear them out thoroughly. It may be of advantage to divide the mass in the middle, turning one half upwards and the other downwards. In tuberculous adenitis there is not the same objection to separating a chain of glands as in malignant disease. Besides these glands along the jugular—the concatenate chain—there may also be tuberculous glands in the posterior triangle of the neck.

Leaving for a time the lower mass of glands, and proceeding to deal with the dissection of the upper set, we come across the bugbear of this operation, the spinal accessory nerve, which passes in a fascial tunnel right through the midst of these glands to traverse the sternomastoid and gain the posterior triangle. Having discovered the nerve, usually between the mass of glands and the edge of the muscle, the roof of the fascial tunnel is slit up so as to enable one to lift it up and push up the glands from beneath it. One may then turn one's attention to the lower part of the wound, and strip down the glands by blunt dissection towards the root of the neck. If there is any difficulty with the lowest glands in the posterior triangle it is a good plan to make a second transverse incision over them.

As regards the suturing of the wound, the essential point is to avoid all tension. The ideal method is first of all to arrest all hæmorrhage—and if this is not possible to use a drainage tube—and to stitch the deep fascia and platysma with a few interrupted sutures. Then to go on to sew the skin edges together, for which many surgeons use Michel's clips; these are a most excellent appliance so long as the patient is not restive, but in those who will not keep still they often result in turning in the edges. For this reason I prefer to use fine horsehair, passed through the edge of the skin only. On this I put a layer of gauze moistened in perchloride of mercury, then a large dressing of sterilised gauze and salicylic wool, using enough to keep the patient's head pretty firmly fixed. For the purpose of pressing the sides of the wound together the inclusion of a marine sponge—an article which, in my opinion, has fallen into quite undeserved disrepute—among the dressings is most valuable, especially when dealing with children, who are so often restless.

### Complications During Operation.

There are several complications that may happen when carrying out this operation. In turning the mass of glands forward the lingual or facial veins may get torn, and there may then be most alarming hæmorrhage. There is, however, no need to be much disturbed when this happens; pack a sponge into the place from which the blood is oozing, continue the operation, and then tie the veins afterwards. If the jugular should be wounded it is better to take out one or two inches of the vein altogether rather than attempt to suture the rent itself. Branches of the facial nerve are very frequently divided, which leads to a drooping of the lip for a few weeks; but I have never seen it last permanently. To wound the spinal-accessory is not necessarily a disaster, though one should, of course, avoid doing so if possible. This is a nerve to be treated with respect; and you must, if you divide it, search for the ends and suture them together. The only other complication is wounding of the thoracic duct, but it is now established that this is not necessarily followed by serious results. There is no objection to ligaturing the thoracic duct if the wound in it cannot be closed. More often the accident is not discovered until a lymph fistula forms; it may then be advisable to reopen the wound and pack it thoroughly.

Let us now consider the gland which has gone on to softening and has thus formed a tuberculous abscess. To remove the glands and the abscess cavity *in toto* is the ideal treatment; but it is often very difficult to carry out, and it is generally better to treat it as a localised abscess; that is to say, to open and drain it, removing the main mass subsequently. In dealing with a very small abscess one may enclose an elliptical piece of the overlying skin by two incisions, and then get rid of the disease by incising the whole mass.

If the abscess bursts the best thing to do is to remove all the cheesy material, drain and dress with antiseptic dressing until it heals; this is often quite successful. Fistulous tracks are often left after an abscess has burst or been drained. These may be dealt with by first packing them with a small strip of gauze soaked in pure carbolic acid. Then an incision is made enclosing the skin surrounding the fistula, and two or three sutures are put through so as to cover in the opening. After again cleaning the skin, and with a new knife the surgeon then endeavours to dissect out the fistulous track entire and to get a clean wound which he can sew up and which will heal by first intention.

